# Differential Effects of Tai Chi Chuan (Motor-Cognitive Training) and Walking on Brain Networks: A Resting-State fMRI Study in Chinese Women Aged 60

**DOI:** 10.3390/healthcare8010067

**Published:** 2020-03-24

**Authors:** Chunlin Yue, Yanjie Zhang, Mei Jian, Fabian Herold, Qian Yu, Patrick Mueller, Jingyuan Lin, Guoxiang Wang, Yuliu Tao, Zonghao Zhang, Liye Zou

**Affiliations:** 1 Department of Physical Education, Soochow University, Suzhou 215021, China; y_chunlin002@163.com (C.Y.); JianMei_Zp@163.com (M.J.); kwang63@163.com (G.W.); Taoyuliu@suda.edu.cn (Y.T.); 2 Exercise and Mental Health Laboratory, Shenzhen University, Shenzhen 518060, China; elite_zhangyj@163.com (Y.Z.); yuqianmiss@163.com (Q.Y.); linjingyuan921@126.com (J.L.); 3 Health and Exercise Science Laboratory, Institute of Sports Science, Seoul National University, Seoul 100744, Korea; 4 Research Group Neuroprotection, German Center for Neurodegenerative Diseases (DZNE), Leipziger Str. 44, 39120 Magdeburg, Germany; Fabian.herold@dzne.de (F.H.); Patrick.Mueller@dzne.de (P.M.); 5 Department of Neurology, Medical Faculty, Otto von Guericke University, Leipziger Str. 44, 39120 Magdeburg, Germany

**Keywords:** exercise, tai chi, ICA, brain function, cognition

## Abstract

*Background:* This cross-sectional study aimed to investigate whether a long-term engagement in different types of physical exercise may influence resting-state brain networks differentially. In particular, we studied if there were differences in resting-state functional connectivity measures when comparing older women who are long-term practitioners of tai chi chuan or walking. *Method:* We recruited 20 older women who regularly practiced tai chi chuan (TCC group), and 22 older women who walked regularly (walking group). Both the TCC group and the walking group underwent a resting-state functional magnetic resonance imaging (rs-fMRI) scan. The acquired rs-fMRI data of all participants were analyzed using independent component analysis. Age and years of education were added as co-variables. *Results:* There were significant differences in default network, sensory-motor network, and visual network of rs-fMRI between the TCC group and walking group (*p* < 0.05). *Conclusions:* The findings of the current study suggested that long-term practice of different types of physical exercises (TCC vs. walking) influenced brain functional networks and brain functional plasticity of elderly women differentially. Our findings encourage further research to investigate whether those differences in resting-state functional connectivity as a function of the type of physical exercise have implications for the prevention of neurological diseases.

## 1. Introduction

Cognitive impairment is one of the greatest health threats nowadays, leading to loss of ability to perform daily activities and poor quality of life among the older adults [1,2]. Most adults experience cognitive decline with advancing age [3,4,5,6,7], and a decline in cognitive performance that exceeds the normal age-related range is associated with neurological diseases such as dementia and Alzheimer’s disease [8,9]. Dementia and Alzheimer’s disease have become common chronic diseases in China [8,9]. Interestingly, there is solid evidence in the literature that reveals that there are considerable changes in resting-state functional connectivity (e.g., in default mode network (DMN)) in neurological diseases such as Alzheimer’s disease [10,11,12,13,14,15,16]. These changes in the resting-state functional connectivity are important because they are linked to changes in cognitive performance [13,14,17,18]. Such changes in resting-state functional connectivity seem also to be of high relevance with regard to tasks of daily living (e.g., mobility) because in individuals suffering from mild cognitive impairments (MCI) it has been observed that changes in default mode network and sensory-motor network connectivity are associated with changes in life-space mobility [19].

In order to prevent neurological diseases such as MCI and Alzheimer’s disease, it is recommended that life style factors should be changed, with a leading role being attributed to the increase of habitual physical activity levels [20]. An increase in habitual physical activity levels is typically engendered through physical training which, in general, promotes an increase in physical fitness and positive influence upon neurocognition [21]. With regard to resting-state functional connectivity measures, previous research has revealed that a higher level of cardiorespiratory fitness is linked to increased DMN and the increased level of functional connectivity in DMN mediates the relationship between cardiorespiratory fitness level and cognitive performance [22]. Furthermore, it has also been observed that a 12 week walking training increased functional connectivity in individuals with mild cognitive impairment [23]. The mentioned evidence suggests that physical training (e.g., aerobic exercise such as walking) that, in general, fosters higher levels of fitness (e.g., cardiorespiratory fitness) is beneficial to improve functional connectivity and, in turn, might help to prevent or delay the manifestation of Alzheimer’s disease. Interestingly, there is also evidence in the literature showing that motor-cognitive training (e.g., tai chi chuan) improves cognitive function to a greater extent than purely aerobic exercises such as walking; however, thus far, how a long-term training of motor-cognitive exercise (e.g., tai chi chuan) influences parameters of brain functioning (e.g., resting-state functional connectivity) has been relatively sparsely investigated [24].

Tai chi chuan (TCC) is an Eastern form of multicomponent exercise that combines aerobic exercise, constant attention, deep breathing, and relaxation. The characteristics of TCC are gentle, lingering movement and unity of body and mind. Hence, TCC appeals to a larger number of older adults in terms of practice. Nowadays, TCC has been widely recognized around the world. According to the National Institutes of Health statistics, 2.3 million American adults were practicing TCC in the past year [3]. Walking is a safe, convenient, and practical exercise that is attracting a larger number of individuals worldwide. Moderate walking can promote many benefits to the body, such as the use of glucose in blood, increasing the elasticity of arteries, improving peripheral blood circulation, eliminating blood flow deposition in tissues, and maintaining a good mood in adults [25]. Brain imaging studies have shown that physical or social activities are beneficial in improving cognitive abilities and optimizing brain structure and function in older adults [26]. However, the exact mechanisms of how TCC and walking exercise may differentially influence the human brain and its function is relatively unknown. Given the importance of resting-state connectivity as parameter of brain functioning [27], the aim of this study was to investigate the influence of long-term TCC training and walking training on resting-state functional connectivity measures, which may foster our understanding of the effects of long-term practice of different types of physical exercises on brain function and explain the beneficial effects of TCC on cognitive functions [24,28].

## 2. Methods

### 2.1. Study Population

In this study, we used the same sample published in previous study, but with different aims [29]. Specifically, 46 older women, from Suzhou city, China, were recruited as participants for this study. Inclusion criteria were normal hearing, no serious physical illness, no family history of mental illness, and long-term tai chi or walking. Exclusion criteria included mental disorder, alcoholism, drug abuse, musculoskeletal impairment, or neurological disorders. Of the population, three participants were excluded due to severe claustrophobia (*n* = 1) and high head motion (*n* = 2). Finally, 42 participants met the eligibility criteria. Eligible participants were informed about this trial (e.g., the purpose of study, study content) prior to enrollment in the study. All participants who volunteered to engage in this study completed the written informed consent form, which was approved by the ethics committee of the university (approval no. ECSU-2019000209). Furthermore, all study procedures were in accordance with the latest version of the Declaration of Helsinki.

### 2.2. Functional Magnetic Resonance Data Acquisition

Data were acquired using a 3T whole body scanner (PHILIPS Ingenia) in the imaging center, the second affiliated hospital of Soochow University. Functional images were acquired using an gradient echoplanarimagine (EPI), with the following scan parameters: TR = 2000 ms, TE = 30 ms, field of view (FOV) = 220 mm × 220 mm, flip angle (FA) = 90°, matrix size = 64 × 64, number of slices = 36, slice thickness = 4 mm, and the total acquisition time for each sequence was 400 s. For each participant, a low noise template T1 was generated as follows: (a) the bias-corrected and intensity-normalized images were upregulated to 0.5 mm^3^ with trilinear interpolation; (b) an initial mean image was calculated, and brainmask generated by affine transforming the T1 brainmask into this space; (c) each interpolated image was rigid-registered to the mean (from its original orientation); (d) another mean was then calculated. The last two steps (c,d) were repeated four times and the final mean constituted the “template” for that subject. Each participant was required to keep their head and body still during the scan period, and all scanning operations were performed by radiologists skilled in magnetic resonance imaging. 

### 2.3. Data Prepossessing

Analysis of the resting-state functional magnetic resonance imaging (fMRI) data were performed using DPABI 4.3 toolbox in Matlab 13b platform. The prepossessing included the following procedures: (1) data format conversion; (2) removal of the first 10 volumes; (3) slice timing correction; (4) head motion correction—each participant’s head motion was below 3 mm and 3°; (5) spatial normalization to standard Montreal Neurological Institute (MNI) space, and isotropic voxels were 3 mm × 3 mm × 3 mm; (6) smooth processing—an 8 mm × 8 mm × 8 mm full width at half maximum Gaussian kernel was used to smooth the normalized images. 

### 2.4. Group Independent Component Analysis

**Figure 1:**
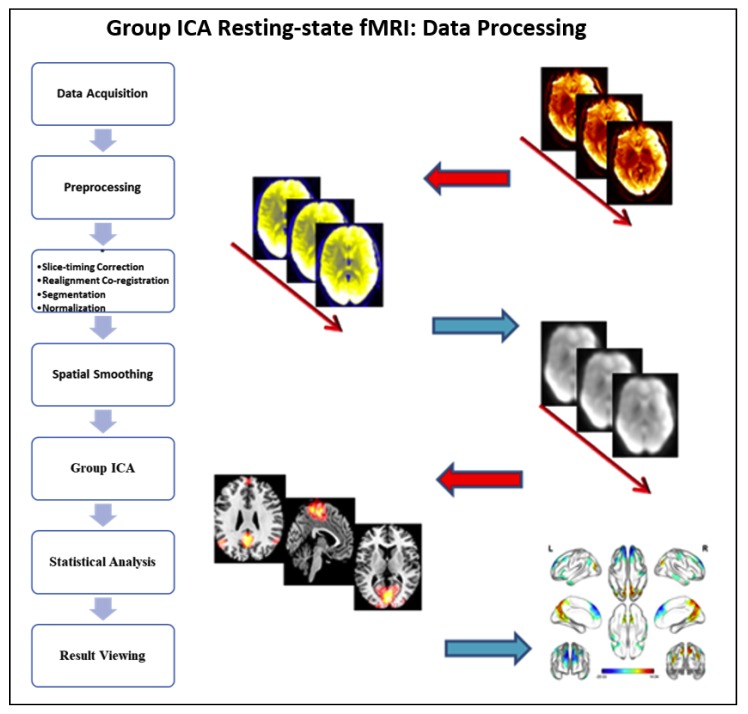
General flowchart of independent component analysis.

Independent component analysis (ICA) was conducted to analyze the preprocessed fMRI data in GIFT (group ICA of fMRI toolbox) (Figure 1) [30,31]. The ICA method did not need to make model assumptions in advance. First, the observation data of all participants were combined according to time to form an observation matrix, and then the ICA algorithm was used to decompose the observation matrix into a mixture matrix and a spatial independent component matrix. The mixture matrix was split by time. The ICA time series of each subject could be obtained, and finally, the ICA map corresponding to each ICA time series could be obtained by back reconstruction [32]. According to the mean images of independent components of all subjects, as well as the ICA independent component template from Smith’s study, spatial multiple regression was performed [33]. On the basis of the regression value of each independent component, the functional network of resting state in TCC and walking group were obtained by selecting the most matched template in the default mode network (DMN), sensory-motor network (SMN), and visual network (VN).

### 2.5. Statistical Analysis

The statistical software SPSS 19.0 (IBM SPSS Inc., IL, USA) was used to conduct the independent sample *t*-test on age and education years between the TCC group and walking group. All *p*-values <0.05 were considered statistically significant. All measured values were expressed as mean ± standard deviation. For image preprocessing, the single sample *t*-test within the group and the independent sample *t*-test between the groups were used for statistical analysis in the statistical parametric mapping 12 (SPM 12) software (Statistical Parametric Mapping, Wellcome Department of Cognitive Neurology, London, United Kingdom: http://www.fil.ion.ucl.ac.uk/spm). Familywise error (FWE) correction was used in the group and the voxel size was more than 20. The union set of two groups of single sample *t*-test results was considered as the comparison range of the two groups of brain functional maps. FWE correction was applied between groups, and the brain regions were defined as statistically significant differences when the voxel size was more than 20. The results were presented using xjview and BrainNet viewer. 

## 3. Results

### 3.1. Subject’s Characteristics

Participants’ characteristics in the present study are presented in Table 1. There were 20 professional female TC practitioners in the TCC group. Participants were on average 62.9 ± 2.38 years old, and had 9.05 ± 1.8 years of education. They practiced Yang style TCC 4 ± 1 times weekly for about 1.5 h each time for more than 6 years. A total of 22 females that were 63.27 ± 3.58 years old were included in the walking group, who had received 8.86 ± 2.74 years of education. For more than 6 years, they mainly exercised by walking, not less than 5 times a week, with each time being no less than 1.5 h. There were no significant differences in age (*p* = 0.193), years of education (*p* = 0.074), mini-mental state examination (MMSE) score (*p* = 0.636) and Montreal Cognitive Assessment (MoCA) score (*p* = 0.83) between the TCC group and walking group.

### 3.2. ICA between TCC Group and Walking Group

**Figure 2:**
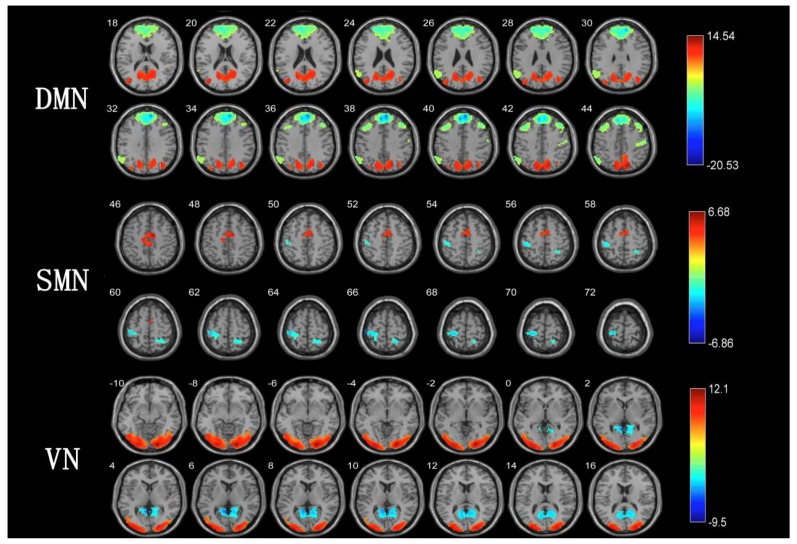
Different results of brain network between TCC group and walking group. Note: Figure 2 is the different results of brain network between TCC group and walking group. Red represents the brain area of the TCC group greater than that of the walking group, blue represents the brain area of the walking group greater than that of the TCC group, and the color bar on the right represents the *t*-value. *p* < 0.05, voxel number >20. The results DMN (default mode network), SMN (sensory-motor network), and VN (visual network) were corrected by family-wise error.

We observed a significant difference in DMN between the TCC group and walking group (Table 2, Figure 2) even after FWE correction. Compared to the walking group, long-term TCC training can enhance the functional connection of precuneus and middle occipital gyrus. In contrast, long-term walking training can enhance the functional connections of the middle temporal gyrus, medial prefrontal lobe, and angular gyrus. 

There were significant differences in SMN between the TCC group and walking group (Table 2, Figure 2) that remained statistically significant even after adjusting for multiple comparisons using false discovery rate (FDR) correction. The TCC group had greater activation in the left supplementary motor area and the right inferior occipital gyrus than the walking group. In contrast, in the walking group, the activation degree of the right posterior center and the right superior loop was higher than that in the TCC group.

Furthermore, even after FWE correction, the differences in VN between the TCC group and walking group remained statistically significant (Table 2, Figure 2). The activation degree of the left and right occipital middle gyrus in the TCC group was greater than that in the walking group. However, the activation degree of the calcarine fissure and surrounding cortex in the walking group was greater than that in the TCC group.

## 4. Discussion

This resting-state fMRI study was performed to investigate the differences in default network, sensory-motor network and visual network between TCC group and walking group through using the ICA method. The findings from this study suggested that participants in the TCC group and walking group had significant differences in default mode network, sensory-motor network, and visual network of rs-fMRI.

The default network is associated with selective retrieval and classification of episodic memory [34], and plays an important role in self-awareness, self-cognition, and other aspects. The precuneus and posterior cingulate gyrus, as the key brain regions of the default network, mainly contribute to visual-spatial imagination, self-referential processing, and autobiographical memory. The frontal lobe is particularly complex in the brain, involving in a variety of higher cognitive functions (e.g., memory and emotion), whereas the interconnections and interactions between the prefrontal lobe and other brain regions of DMN are involved in more complex cognitive processing [35]. The effectiveness of the network may become fragile when multiple networks interact effectively to maintain a high level of cognitive function, such as executive function. The medial prefrontal cortex, a sub-region of the prefrontal cortex, plays a key role in higher brain functions such as emotion processing, working memory, and behavior planning. Previous studies showed that 6 months of aerobic exercise can enhance the functional connectivity in the DMN and in the frontal parietal network, whereas 12 months of aerobic exercise enhanced the functional connectivity between the prefrontal cortex and the temporal cortex, the posterior cingulate cortex (PCC) and the middle temporal gyrus, and the middle temporal gyrus and the parahippocampal place area, thus elevating the functional efficiency of the resting-state default network in older adults [36]. Six months of aerobic exercise can also increase the volume of the prefrontal cortex, selectively affecting the functional connectivity between the DMN prefrontal lobe and middle temporal gyrus, delaying cognitive decline [37]. In our study, although the activation level of the medial prefrontal cortex in the TCC group was lower than that in the walking group, the activation level of the precuneus functional connectivity in the TCC group was greater than that in the walking group in the resting-state. This suggested that long-term TCC exercise had a greater effect on the function of precuneus. Our study also found that the activation level of DMN in the TCC group was greater than that in the walking group. Given that the DMN mediates the effects of cardiorespiratory fitness on cognition, it seems reasonable to speculate that the increased DMN functional connectivity in the TCC group may explain, at least partly, the effect of practicing TCC on delaying cognitive aging [22]. However, this assumption requires further investigations.

The central posterior gyrus, as the highest center of the somatosensory system, receives shallow and deep sensations from the whole body. Changes in the central posterior gyrus directly affect the sensory function of the human body. In addition, the central posterior gyrus also plays an important role in attention function. The central posterior gyrus can regulate the attention reorientation, especially when the target is repositioned [38]. Although the activation levels of the right posterior center and right parietal gyrus of the TCC group were smaller than that in the walking group, the functional connectivity of the left supplementary movement area and the right inferior occipital gyrus in the TCC group was greater than that in walking group in the resting state. This current study suggested that, compared with walking exercise, long-term TCC exercise may promote sensory-motor activation. Relatedly, Burzynska et al. have investigated functional connectivity differences of 20 expert dancers within the motor learning network as compared to 20 age- and education-matched non-dancers, suggesting that expert dancers showed greater activation of the action observation network related to dance skill and balance [39]. The occipital gyrus, as an important brain region of the visual network, plays an important role in processing auditory and tactile stimuli, especially related to the accuracy of individual sound localization performance [40]. Our study showed that the visual network activation of the TCC group was greater than that of the walking group, suggesting that regular TCC exercises may increase the activation level of the left and right lateral midoccipital gyrus.

TCC, as a moderate intensity, slow-paced, and rhythmic exercise, is gaining increasing attention as a potentially effective exercise method to improve brain health and delay brain aging [41]. Earlier studies have found that TCC may be an effective way to prevent brain volume loss and improve cognitive function [42,43,44,45]. Pieramico et al. pointed out that multi-modal exercise can be an effective strategy to prevent aging-related cognitive loss, and significant capability of functional and structural changes were maintained in older adults [45]. Other studies have shown that regular physical training may contribute to changes in brain structure [46], brain function [47,48], and alleviate age-related cognitive decline [49]. In the majority of exercise cognition imaging studies, researchers have focused on short-term effects rather than prolonged cognitive benefits of exercise, particularly for motor-cognitive exercise training such as tai chi chuan that is culturally suitable for Chinese populations. An important feature of aging is cognitive change, which changes with brain structure and function. Our study found that TCC exercise may be a potential regulator of brain aging, which can delay the decline of the nervous system during human aging. TCC is also expected to be an effective intervention approach to delay the aging changes in brain structure and to benefit physical and mental health. Furthermore, there are considerable changes in resting-state functional connectivity in Alzheimer’s disease, and on the basis of our findings that show that TCC has a positive effect on measures of resting-state functional connectivity, we can speculate that TCC could be a promising intervention strategy to prevent or delay the onset of this neurological disease [10,11,12,13,14,15,16].

Although it is well known that cognitive ability declines with aging, there were many controversial issues around the underlying mechanism of cognitive decline. On the basis of the continuous development of brain imaging technology, further studies are needed to explore the brain mechanism on the effects of TCC on the brain function among older adults through multimodal magnetic resonance analysis methods (e.g., effective connectivity, fiber tracking, and dynamic causal models). Using the combination of these different research methods will allow us to better understand the effects of TCC on the brain comprehensively. 

The findings of the present study should be interpreted by considering the following limitations. In the current study, we recruited a relatively small but homogenous sample of right-handed older women to avoid gender effects and laterality effects. Hence, on the basis of our sample, the current findings cannot be generalized to older men. Future studies that aim to investigate the cognitive (neurological) benefits of long-term TC training should also focus on older men in order to examine the influence of sex. 

## 5. Conclusions

Our findings suggested that TCC and walking exercise had different effects on brain networks in DMN, SMN, and VN, and may have different effects on brain functional plasticity in older adults. Given that there are considerable changes in brain networks in neurological diseases such as Alzheimer’s disease, our findings encourage further studies to investigate whether long-term TCC training interventions may prevent or delay the onset of such neurological diseases.

## Figures and Tables

**Table 1:** Characteristics of study participants.

	Tai Chi Chuan	Walking	*t*	*p*
	*n* = 20	*n* = 22		
Age (year)	62.9 ± 2.38	63.27 ± 3.58	−0.393	0.193
Education (years)	9.05 ± 1.8	8.86 ± 2.74	0.188	0.074
Handedness (left/right)	0/20	0/22	−	−
MMSE	28.5 ± 1.1	28.14 ± 1.0	1.1	0.636
MoCA	28.4 ± 1.5	27.5 ± 1.5	1.94	0.83

Abbreviations: MMSE = mini-mental state examination; MoCA = Montreal Cognitive Assessment.

**Table 2:** Comparison of brain network between tai chi chuan (TCC) group and walking group.

Brain Area(AAL)	Voxel Number	Maximum Difference Point MNI Coordinates			*t*-Value at Peak Point
		X	Y	Z	
Default mode network					
Precuneus	1678	−6	−66	60	14.5392
Middle occipital gyrus	144	−36	−75	18	8.9566
Middle temporal gyrus	103	45	3	−45	−7.3819
Medial prefrontal cortex	1957	6	51	39	−20.5336
Angular gyrus	268	−57	−60	45	−9.2211
Sensory-motor network					
Right inferior occipital gyrus	28	42	−78	−12	4.9046
Right supplementary motion gyrus	210	9	−15	36	6.6679
Left postcentral gyrus	177	−48	−27	66	−6.864
Right superior gyrus	73	21	−51	66	−4.999
Visual network					
Left midoccipital gyrus	1099	−24	−78	−15	11.4121
Right midoccipital gyrus	1288	18	−90	−9	12.0978
Calcarine fissure and surrounding cortex	443	−6	−51	3	−9.4966

Notes: *t*-value at peak point is the most significant difference in this brain region; the Montreal Neurological Institute (MNI) coordinates were calculated though the Montreal Institute standard template space.
